# A cross-sectional survey on the lung ultrasound training and practice of respiratory therapists in mainland China

**DOI:** 10.1186/s12890-022-02213-6

**Published:** 2022-11-18

**Authors:** Kai Liu, Yu-long Yao, Yu-xian Wang, Bi-lin Wei, Liu-cun Li, Qi-xing Wang, Hui-qing Ge, Er-shan Wang, Li-min Yang, Huan Chen, Yun-qing Yang, Hao Qin, Wen-jun Zhai, Shen-ji Yu, Xiao-ting Wang, Zhe Luo, Guo-wei Tu

**Affiliations:** 1 Department of Critical Care Medicine, Zhongshan Hospital, Fudan University, Shanghai, 200032 China; 2 Department of Critical Care Medicine, School of Medicine, Renji Hospital, Shanghai Jiao Tong University, Shanghai, China; 3 Department of Critical Care Medicine, The First Affiliated Hospital of Sun Yat-Sen University, Guangzhou, China; 4 Department of Pulmonary and Critical Care Medicine, The Second Xiangya Hospital of Central South University, Changsha, China; 5 Department of Critical Care Medicine, Shanghai Tenth People’s Hospital, Tongji University, Shanghai, China; 6 Department of Respiratory Care, Sir Run Run Shaw Hospital, Zhejiang University School of Medicine, Hangzhou, China; 7 Department of Critical Care Medicine, Peking Union Medical College Hospital, Peking Union Medical College, Chinese Academy of Medical Sciences, Beijing, China; 8 Department of Critical Care Medicine, West China Hospital, Sichuan University, Chengdu, China; 9 Department of Respiratory and Critical Care Medicine, Shanghai Changhai Hospital, Second Military Medical University, Shanghai, China; 10 Department of Critical Care Medicine, School of Clinical Medicine, Beijing Tsinghua Changgung Hospital, Tsinghua University, Beijing, China; 11 Department of Critical Care Medicine, Zhongshan Hospital, Fudan University, Xiamen BranchXiamen, 361015 China; 12 Shanghai Key Lab of Pulmonary Inflammation and Injury, Shanghai, China

**Keywords:** Lung ultrasound, Mainland China, Practice, Respiratory therapist, Training

## Abstract

**Purpose:**

This national study aimed to investigate the lung ultrasound (LUS) training and practice of respiratory therapists (RTs) in mainland China.

**Methods:**

A cross-sectional multicenter survey was conducted from May 22, 2021 to August 12, 2021, through online platforms. This survey included RTs in mainland China. The survey was divided into four sections: (1) demographic characteristics and basic information; (2) basic information about LUS training and practice; (3) LUS practice details; and (4) Other ultrasound training and practice.

**Results:**

A total of 514 responses were received, and 494 valid responses were included in the analysis. 81.2% (401/494) participants’ highest degree of education was a bachelor’s degree, and 43.1% (213/494) participants were at level II in terms of job ranking. 99.2%(490/494) participants agreed that the RTs needed to learn lung ultrasound, but only 12.3% (61/494) participants had received a LUS training course. Further, 66.2% (327/494) experienced participants responded to Sect. 3. Most of RTs used LUS when the patient had hypoxia (265/327, 81%) or dyspnea (260/317, 79.5%); they also used it during spontaneous breathing trial(SBT) (191/327, 58.4%) or in prone position (177/327, 54.1%). The A-line (302/327, 92.4%), B-line (299/327, 91.4%), lung slide (263/327, 80.4%), and bat sign (259/327, 79.2%) were well known as LUS signs. Also, 30.6% (100/327) participants did not use the LUS protocol in their clinical practice, and only 25.4%(83/327) participants said they had used LUS scores. Moreover, 55.7% (182/327) participants frequently changed the respiratory therapy strategy according to LUS results.

**Conclusions:**

We should improve the number and workplace of RTs in mainland China in the future. We should also standardize the application of LUS practice and training for RTs in mainland China and establish corresponding certification pathways.

**Supplementary Information:**

The online version contains supplementary material available at 10.1186/s12890-022-02213-6.

## Introduction

Lung ultrasound (LUS) has become increasingly extensive in both adult and pediatric populations with the great development of point-of-care ultrasound over the past two decades [1–3]. LUS can be used for the early detection and management of lung disease, pleural disease, and heart failure, and for guiding related clinical treatment [4–7]. It has the following advantages: noninvasiveness, absence of radiation, portability, repeatability, and dynamic real-time monitoring. Moreover, LUS is superior to chest x-ray in pleural effusion, interstitial syndrome, pulmonary edema, consolidations and pneumothorax, and may be used as an alternative to chest computed tomography in interstitial pneumonia caused by COVID-19, pleural effusion and consolidations[8–10]. Therefore, it has become more important in treating critically ill patients, even patients with COVID-19 [11–13]. Many associations have formulated guidelines to promote the practice of lung ultrasound, recommended standardized LUS training for physicians and nurses, and enhanced clinical quality control [3, 14, 15].

Respiratory therapists (RTs) are professional technicians engaged in respiratory therapy and are important members of the patient's treatment team. Under the guidance of doctors, they use professional methods to prevent, evaluate, diagnose, treat, manage, educate, and care for patients with cardiopulmonary insufficiency or abnormalities. A recent study pointed out the significance of LUS for RTs [16]. However, RTs are less likely to have clinical or ultrasound knowledge than physicians. They have more clinical restrictions and need more standardized training [17].

Respiratory care in mainland China started relatively late until the Run Run Shaw Hospital of Zhejiang University first established the department of respiratory care in 1994, and the current number of RTs is still meager, which is caused by many factors, such as economy, education system and so on. However, during the outbreak of COVD-19, RTs made an outstanding contribution and received widespread attention and recognition [18]. Although some of them have received LUS training and begun to apply LUS in clinical management, the overall development is still unclear [19]. Therefore, we performed a cross-sectional survey on the LUS training and practice of RTs (LUS-RTs) in mainland China. The findings might provide a better understanding of current development and unmet clinical needs.

## Materials and methods

### Study design

This LUS-RT survey was designed and initiated by the China Critical Ultrasound Research Group (CCUSG). We referred to the relevant literature and use non-blinded discussion with experts in the fields of lung ultrasound, intensive care, and respiratory care; and finally formed the survey items to formulate the contents of the survey and ensure accuracy and professionalism. The final survey items were unanimously agreed upon by the members of the research team. The LUS-RT survey was distributed through various social media, RT network platforms, and an online survey tool (Tencent Questionnaire, Tencent, Shenzhen, China). The survey started on May 22, 2021, and ended on August 12, 2021.

We set up 11 liaison officers to be responsible for different regions in mainland China, thus ensuring enough qualified participants in the survey. The answers to open questions were reviewed by three independent investigators (KL, YLY, and YXW). Only when all questions were answered did we treat it as a valid response. Ethics approval was obtained from Medical Ethics Committee of Fudan University Zhongshan Hospital (B2021-540R). Informed consent was obtained from all individual participants.

The study is reported according to the recommendations of the strengthening the reporting of observational studies in epidemiology (STROBE) statements.

### Survey items

The survey was divided into four sections:

- (1) Demographic characteristics and basic information: Name, age, sex, job ranking, the highest degree of education, years of working as an RT, hospital name, location of the hospital, hospital level, department, number of the ultrasound machines in the department, and types of probes in the department (multiple choice question, MCQ).
- (2) Basic information of LUS training and practice: Do you think the RTs need to learn LUS? Have you received LUS training? What is the personal rating of the level of mastery and application of LUS: Are you willing to receive special LUS training? Can your department charge or report for LUS? What is the frequency of using LUS in your clinical work? What is the cause of limiting your use of LUS (MCQ)? Have you ever used LUS in patients with COVID-19? Do you pay attention to LUS-related researches and papers?
- (3) LUS practice details (for experienced RTs only. Participants were treated as experienced RT if they did not choose “never” when answering “The frequency of applying LUS in your clinical work?” in Sect. 2.): When to use LUS (MCQ)? Which probes are commonly used for LUS (MCQ)? Can you apply the BLUE (bedside lung ultrasound in emergency) protocol to the application of LUS? Can you identify these LUS signs (MCQ)? What are the other LUS protocols that you are using (MCQ)? Can you evaluate different LUS regions using semi-quantitative scores? Do you change the respiratory therapy strategy according to LUS results? Does the clinician approve of your change according to LUS results? Will you issue an official report on the results of LUS? Do you evaluate diaphragmatic dysfunction by diaphragmatic ultrasound (e.g., diaphragmatic inspiratory excursion, thickness of diaphragm, and thickening fraction)? Do you perform ultrasound-assisted tracheotomy? Do you perform ultrasound-assisted chest drainage?
- (4) Other ultrasound training and practice: Do you think the RTs need to learn cardiac ultrasound? Have you received cardiac ultrasound training? Have you mastered and applied other ultrasound (MCQ)?

Detailed questionnaire information is available in Additional file 1: Survey.

### Statistical analysis

The results were expressed as means (standard deviation) or medians for quantitative variables or by the frequency of distribution for qualitative variables. Confidence intervals (CI) of 95% were calculated using the Wilson Score interval, and a two-sided *P* value of 0.05 indicated a statistically significant difference.

## Results

### Study participants

**Fig. 1:**
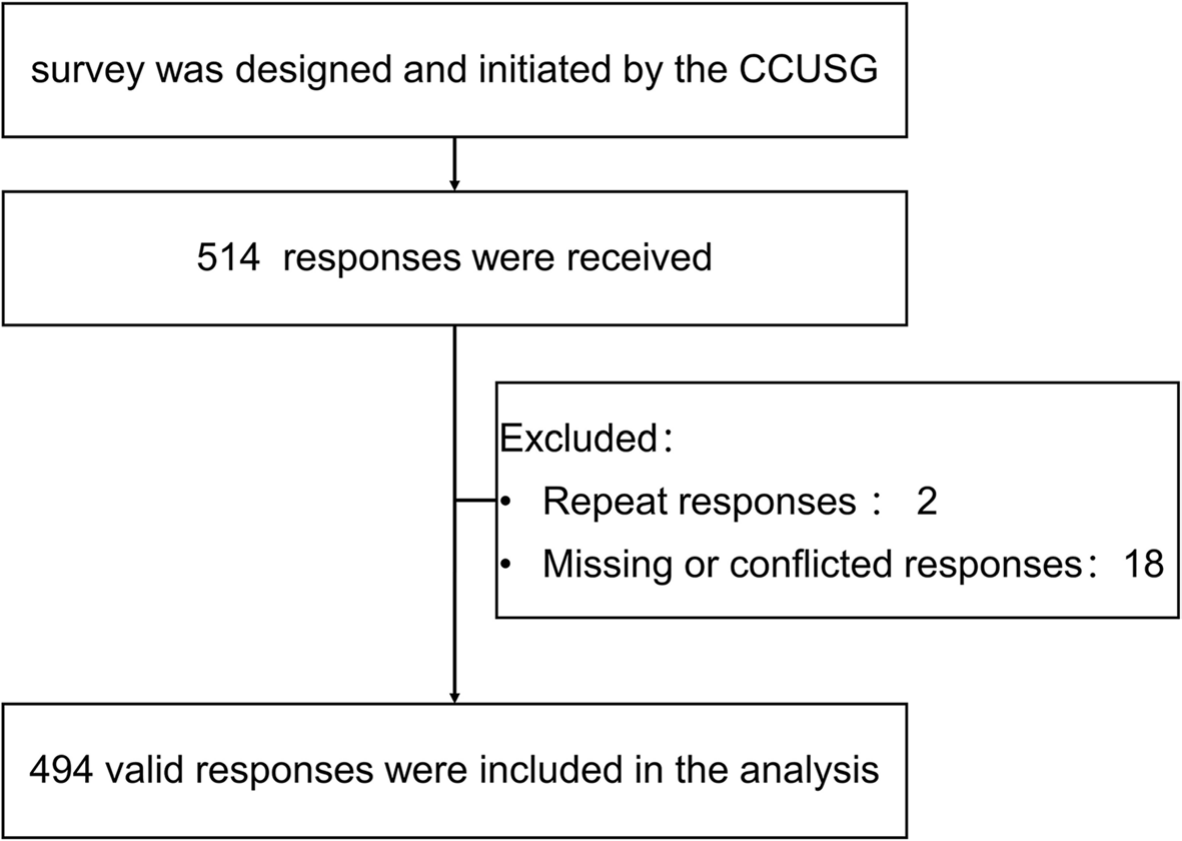
Flow chart. CCUSG, China Critical Ultrasound Study Group

A total of 514 responses were received, of which two were duplicates and 18 were missing or conflicting. The invalid sets of questionnaire were excluded. Finally, 494 valid responses were included in the analysis. The flowchart is depicted in Fig. 1.

### Demographic characteristics and basic information

The characteristics of the 494 survey participants are listed in Table 1.

**Table 1:** Characteristics of the 494 survey participants

Characteristic	
Age, yr, mean ± SD	31.8 ± 6.9
Female, n (%)	248 (50.2%)
The highest degree of education, n (%)	
Associate degree	63 (12.7%)
Bachelor’s degree	401 (81.2%)
Master’s degree	21 (4.2%)
Doctor’s degree	9 (1.8%)
Job ranking, n (%)	
No job rank	51 (10.3%)
Level I	213 (43.1%)
Level II	182 (36.8%)
Level III	43 (8.7%)
Level IV	5 (1.0%)
Years of working as an RT, yr, mean ± SD	5.48 ± 4.46
Level of hospital, n (%)	
Level I	0
Level II	48 (9.3%)
Level III	446 (91.7%)
Department, n (%)	
Respiratory care	33 (6.7%)
Respiratory/Pulmonary	128 (25.9%)
ICU	278 (56.3%)
PICU	12 (2.4%)
NICU	17 (3.4%)
Emergency	20 (4.0%)
Others	6 (1.2%)
Number of the ultrasound machine in department, n (%)	
0	65 (13.2%)
1	250 (50.6%)
2	102 (20.6%)
3	29 (5.9%)
4	20 (4.0%)
≥ 5	28 (5.7%)
Types of probes in department (multi-choice), n (%)	
Curvilinear probe	403 (81.6%)
Linear probe	390 (78.9%)
Phased array probe	372 (75.3%)
Transesophageal ultrasound probe	67 (13.6%)

*RT* respiratory therapist, *ICU* intensive care unit, *PICU* pediatric intensive care unit, *NICU* neonatal intensive care unit

The average age of the participants was 31.8 ± 6.9 years, and 50.2% (248/494) were female. Most of the participants’ (401/494, 81.2%) highest degree of education was a bachelor’s degree. Also, 43.1% (213/494) participants were at level II and 36.8% (182/494) participants were at level III in terms of job ranking. Overall, the average working experience of participants as RTs was 5.48 ± 4.46 years.

**Fig. 2:**
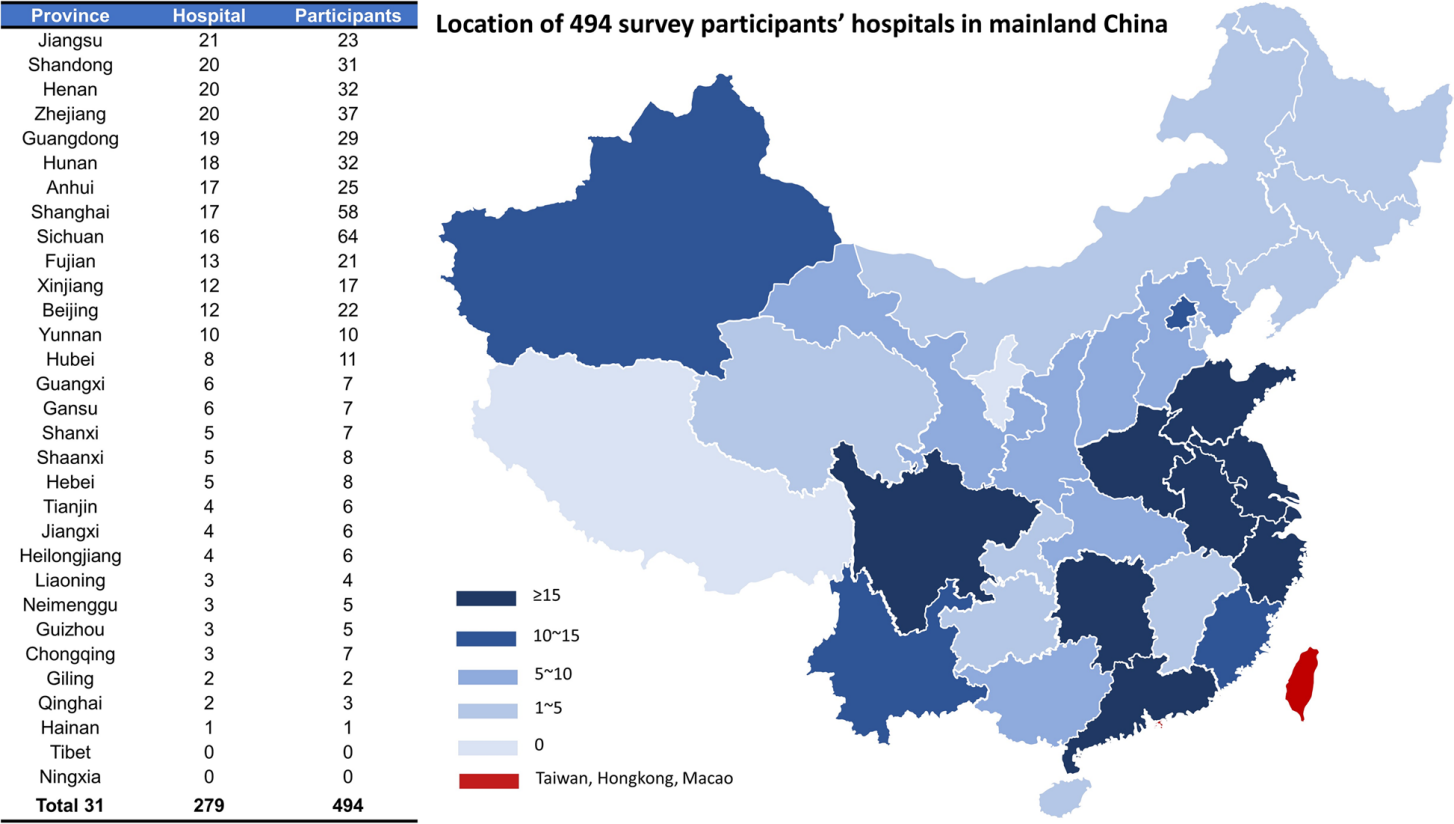
Location of 494 survey participants’ hospitals in mainland China

The hospital level of 91.7%(446/494) participants was level III. The location of the hospital is summarized in Fig. 2. RTs from more than 20 hospitals in Jiangsu, Shandong, Henan, Zhejiang, and Guangdong participated in the survey. Sichuan and Shanghai had the largest number of participants. Moreover, 56.3%(278/494) participants worked in the intensive care unit (ICU), 25.9%(128/494) participants worked in the respiratory/pulmonary department, and 6.7%(33/494) participants worked in the respiratory therapy department.

The information on ultrasound machines in the participant’s department was also provided in Sect. 1. Most participants stated the availability of 1 (250/494, 50.6%) or 2 (102/494, 20.6%) ultrasound machines in their department, and 13.2%(65/494) participants stated that no ultrasound machine was available in their department. The curvilinear probe (403/494, 81.6%), linear probe (390/494, 78.9%), and phased-array probe (372/494, 75.3%) were available in most departments.

### Basic information on LUS training and practice

The basic information on LUS training and practice is listed in Table 2.

**Table 2:** Basic information of LUS training and practice

*Questions*	*n (%)*
Do you think the RTs need to learn LUS?	
Yes	490 (99.2%)
No	4 (0.8%)
Have you received LUS training?	
No training or simple training	158 (32.0%)
In-hospital training	235 (47.6%)
In other hospital training	40 (8.1%)
Special LUS training course	61 (12.3%)
Are you willing to receive special LUS training course?	
Yes	480 (97.2%)
No	14 (2.8%)
Can your department charge and report for LUS?	
Yes	129 (26.1%)
No	365 (73.9%)
The frequency of using LUS in your clinical work	
Never	167 (33.8%)
Rarely	215 (43.5%)
Sometimes	91 (18.4%)
Frequently	21 (4.3%)
The cause of limiting your use of lung ultrasound (multi-choice)	
Lack of proficiency	381 (77.1%)
Lack of time	248 (50.2%)
Lack of machine	195 (39.5%)
Lack of charges	137 (27.7%)
Lack of trust from clinicians	51 (10.3%)
Have you ever used LUS in COVID-19 patient?	
No management	141 (28.5%)
Yes	64 (13.0%)
No	289 (58.5%)
Do you pay attention to LUS related research and papers?	
Yes	311 (63.0%)
No	183 (37.0%)

*LUS* lung ultrasound, *RT* respiratory therapist, *COVID-19* Corona Virus Disease 2019

**Fig. 3:**
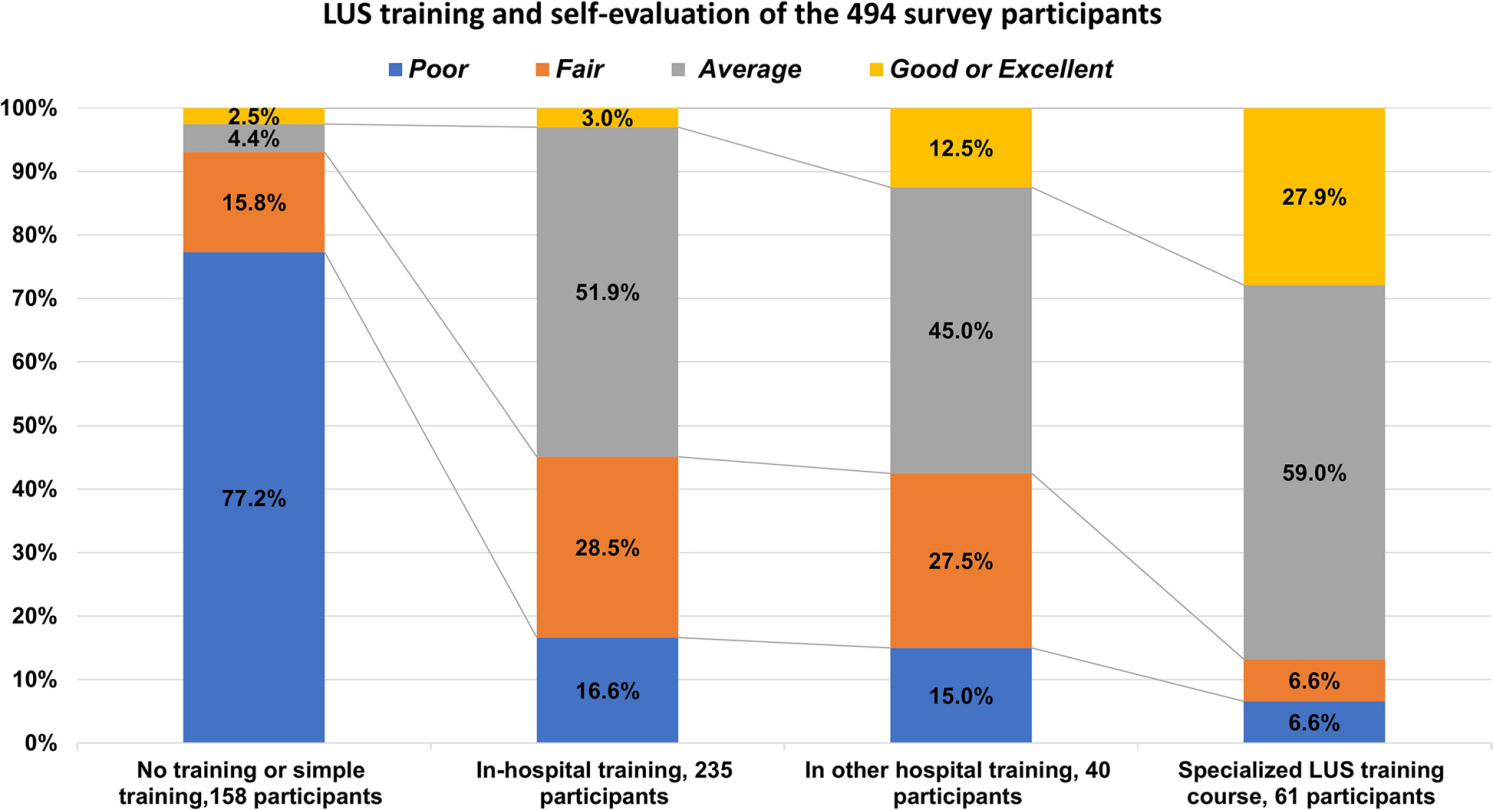
LUS training and self-evaluation of the 494 survey participants. LUS, Lung ultrasound

Nearly all participants (490/494, 99.2%) agreed that the RTs needed to learn LUS. Also, 32.0%(158/494) participants did not receive LUS training. In addition, 47.6% (235/494) participants received LUS training in the hospital they were working at, and 8.1%(40/494) participants received LUS training in a different hospital. Only 12.3%(61/494) participants received a special LUS training course. Different training experiences led to different LUS self-evaluation results. The results are shown in Fig. 3. Among the participants with no training or simple LUS training, 122 (77.2%/158) and 15.8% (25/158) rated their LUS capabilities as poor or fair. In contrast, among participants who received special LUS training, 59% (36/61) rated their abilities as average and 27.9%(17/61) believed that they were excellent. Further, 97.2% (480/494) participants were willing to receive a special LUS training course.

Further, 73.9% (365/494) participants said that LUS practices could not be charged. Also, 4.3% (21/494) and 18.4%(91/494) participants said that they had frequently or sometimes practiced LUS in the clinic, and 43.5% (215/494) participants rarely practiced lung ultrasound. Moreover, 77.1% (381/494) participants said that the lack of proficiency was one cause of limiting the use of LUS practice. Other reasons included lack of time (248/494, 50.2%), lack of machines (195/494, 39.5%), lack of charges (137/494, 27.7%), few participants (51/494, 10.3%), and lack of trust from clinicians. Also, 13.0% (64/494) participants said that they had used LUS in patients with COVID-19.

### LUS practice details

Further, 66.2% (327/494) experienced participants responded to this section of the question. The LUS practice details of the 327 experienced participants in clinical work are listed in Table 3.

**Table 3:** LUS practice details of the 327 experienced participants in clinical work

*Questions*	n (%)
Which probes used for LUS (multi-choice)	
Curvilinear probe	282 (86.2%)
Linear probe	173 (52.9%)
Phased array probe	57 (17.4%)
Can you practice LUS by the BLUE protocol?	
Yes	176 (53.8%)
No	151 (46.2%)
Other LUS protocols that you use (multi-choice)	
Eight Zone Examination	101 (30.9%)
Twelve Zone Examination	82 (25.1%)
Twenty-eight Zone Examination	9 (2.8%)
PLUE Protocol	88 (26.9%)
No Protocol	100 (30.6%)
Can you evaluate aeration in different lung zone by semi-quantitatively LUS score?	
Yes	83 (25.4%)
No	244 (74.6%)
Do you change the respiratory therapy strategy according to LUS results?	
Never	7 (2.1%)
Rarely	31 (9.5%)
Sometimes	107 (32.7%)
Frequently	182 (55.7%)
Does the clinician approve of your change according to LUS results?	
Reject	3 (0.9%)
Rarely accept	42 (12.8%)
Sometimes or mostly accept	220 (67.3%)
Completely accept	62 (19.0%)
Will you issue an official report on the results of LUS?	
Yes	21 (6.4%)
No	306 (93.6%)
Do you evaluate diaphragmatic dysfunction by diaphragmatic ultrasound (eg. diaphragmatic inspiratory excursion, thickness of diaphragm, and thickening fraction)?	
Yes	200 (61.2%)
No	127 (38.8%)
Do you use ultrasound-assisted tracheotomy?	
Yes	66 (20.2%)
No	261 (79.8%)
Do you use ultrasound-assisted chest drainage?	
Yes	205 (62.7%)
No	122 (37.3%)

*LUS* lung ultrasound

**Fig. 4:**
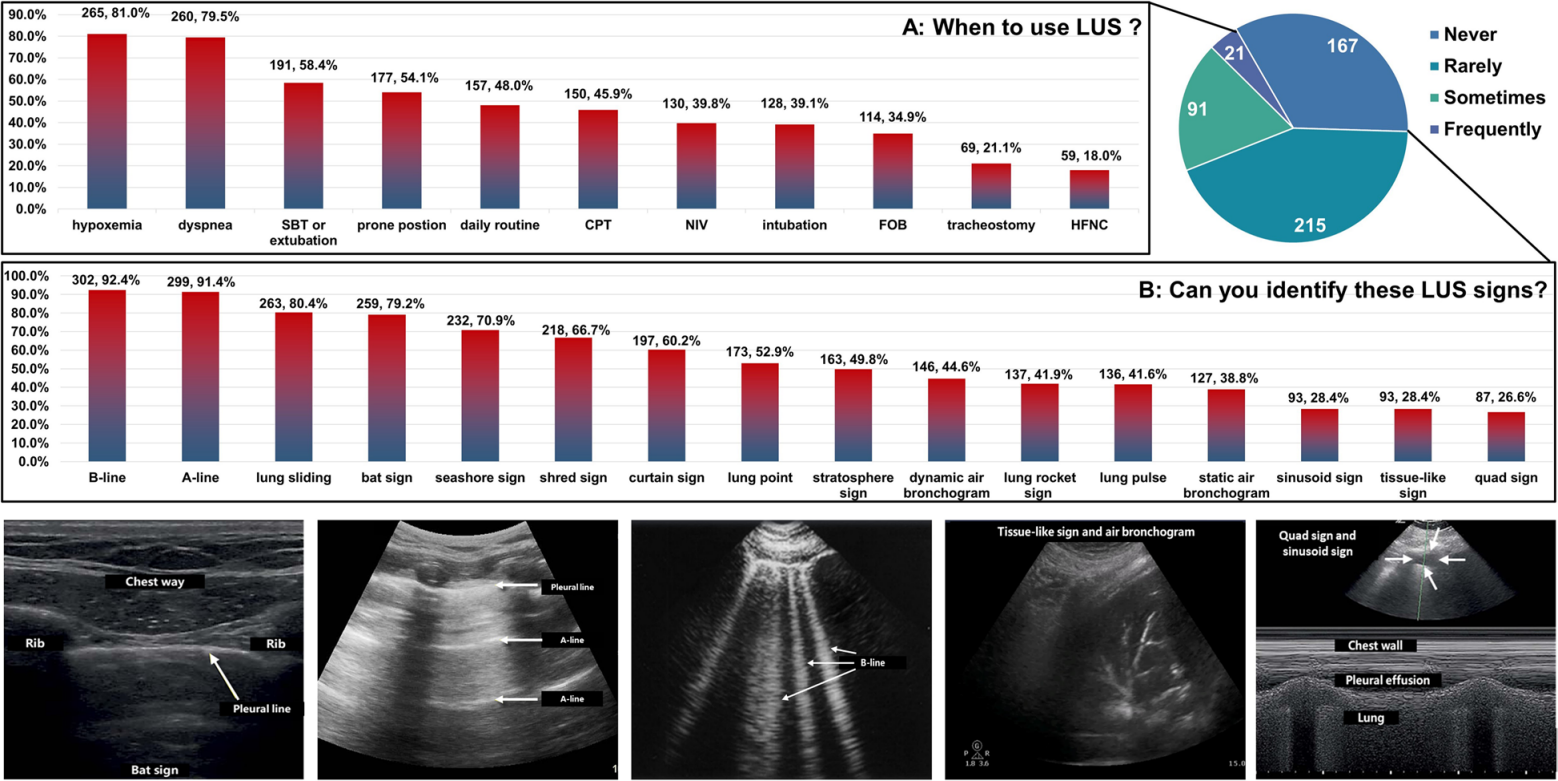
**A** When to use LUS? **B** Can you identify these LUS signs? LUS, Lung ultrasound; SBT: spontaneous breathing trial; CPT: chest physiotherapy; NIV: non-invasive ventilation; FOB fiber bronchoscopy; HFNC: high-flow oxygen therapy

In clinical practice, a large majority of participants 86.2% (282/327) used a curvilinear probe to perform LUS, and 52.9% (173/317) participants used a linear probe. Nearly half of the participants (157/327, 48%) used LUS daily, but more participants used LUS when the patient had hypoxia (265/327, 81%) or dyspnea (260/317, 79.5%); they also used it during spontaneous breathing trial (SBT) (191/327, 58.4%) or in prone position (177/327, 54.1%). A few participants used LUS during other respiratory therapy practices. The details are listed in Fig. 4A. For identifying LUS signs, the A-line (302/327, 92.4%), B-line (299/327, 91.4%), lung slide (263/327, 80.4%), and bat sign (259/327, 79.2%) were well known as LUS signs. In contrast, quad sign (87/327, 26.6%), tissue-like sign (93/327, 28.4%), and sinusoid sign (93/327,28.4%) were rarely known. The details are listed in Fig. 4B.

Moreover, 53.8% (176/327) participants used the BLUE protocol; however, 30.6% (100/327) participants did not use the LUS protocol in their clinical practice. Only 25.4% (83/327) participants said that they used semi-quantitative LUS scores. Also, 55.7% (182/327) participants frequently changed the respiratory therapy strategy according to LUS results; 32.7%(107/327) participants sometimes did so. Further, 93.6%(306/327) participants did not issue an official report on the results of LUS. Also, 61.2% (200/327) participants evaluated diaphragmatic dysfunction by diaphragmatic ultrasound, 20.2% (66/327) participants performed an ultrasound-assisted tracheotomy, and 62.7% (205/327) participants performed ultrasound-assisted chest drainage.

### Other ultrasound training and practice

The details of other ultrasound training and practice are listed in Table 4. Regarding the necessity of learning cardiac ultrasound for RTs, 92.9% (459/494) participants answered yes. Also, 39.1% (193/494) participants received cardiac ultrasound training, whereas 32.2% (159/494) received another ultrasound training.

**Table 4:** Other ultrasound education and practice of the 494 survey participants

*Questions*	*n (%)*
Do you think the RTs need to learn cardiac ultrasound?	
Yes	459 (92.9%)
No	35 (7.1%)
Have you received cardiac ultrasound training?	
Yes	193 (39.1%)
No	301 (60.9%)
Have you received another ultrasound training, like cerebral ultrasound, kidney ultrasound, abdominal ultrasound, transesophageal ultrasound, ultrasound-guided invasive operation (arterial catheterization, deep vein catheterization, etc.)?	
Yes	159 (32.2%)
No	335 (67.8%)

*RT* respiratory therapist

## Discussion

One of the main findings of this survey was that the number of RTs participating in the questionnaire in mainland China far exceeded that in previous studies [19]. RTs are distributed in many provinces, and the largest number of hospitals or participants are in relatively economically developed areas (Jiangsu, Zhejiang, Shanghai, Guangdong, etc.) or areas with respiratory therapy undergraduate schools (such as Sichuan). Most of them have a bachelor's degree and a junior job title. RTs in the New York state had more employment places such as sleep centers, universities, and so forth, while RTs in mainland China were mainly concentrated in the critical care medicine department and respiratory department of the level III hospitals [20]. In addition, more experienced RTs (> 15 years) were found in the New York state, while the vast majority of RTs in mainland China were young [20]. Therefore, the development of respiratory therapy in mainland China is still in an early stage, but full of hope. In only 13.2% (65/494) of the workplaces of RTs currently have no ultrasound machines, and most have multiple probes. This may be due to the continuous promotion of point-of-care ultrasound in recent years.

In this survey, RTs nearly fully acknowledged that they needed to learn LUS. However, currently, only 12.3% (61/494) of them received special training, which is a proportion that needs improvement. As shown in Fig. 3, we found a clear difference in the self-assessment of LUS understanding between RTs with special LUS training and those with no or simple training. This is an important reminder that LUS requires special training, and the results have been confirmed [17, 21–23]. As reported in previous studies, LUS still has many limitations in clinical practice, mainly due to its own factors such as insufficient time and proficiency, and other objective factors such as the lack of machines [24–26]. A multicenter international study indicated that 25 training sessions were required, and 80% of the trainees could master the basic skills and significantly reduced the average duration [21]. In addition, most departments could not issue LUS reports, which also restricted the direct use of LUS by these doctors and RTs, and often required the help of ultrasound specialists [25].

During the COVID-19 outbreak, LUS has received enormous attention due to the limited availability of other lung diagnostic equipment [13, 27]. Studies have shown that LUS can help in early identification and stratification and also guide the management of patients suspected of having COVID-19, outpatient and emergency patients, and hospitalized patients; LUS scores are also related to patient prognosis [10, 28–31]. Different categories of medical staff, such as anesthesiologists, critical care, and even primary care physicians, should master LUS to cope with the stress of the pandemic, which may help speed up resource allocation in special times [22, 32]. Our survey showed that more than 70% of RTs managed patients with COVID-19, and 13% of RTs applied LUS during the period. This rate of use of LUS was similar to the rates reported by Vetrugno and colleagues for managing patients with COVID-19 in various Italian regions [32]. This was an encouraging result, needing further promotion.

The LUS practice details of the 327 experienced participants highlighted the importance of LUS training. We found that the RTs varied widely in these questions. It was very important to quickly diagnose the etiology of acute respiratory failure. Hypoxia and dyspnea were the most frequently used opportunities to conduct LUS, and 53.8% (176/327) of RTs were screened using the BLUE protocol [33]. Some critically ill patients may experience repeated SBT failures, leading to difficulty in weaning, respiratory failure after extubation, or even re-intubation [34]. These patients need to be identified and dealt with as soon as possible. LUS is an effective diagnostic and monitoring tool [35–37]. We found that 58.4% (191/327) participants practiced LUS before and after SBT. The BLUE protocol can be used to predict prone positioning potential and assess prognosis in patients with Acute respiratory distress syndrome (ARDS) [38]. Benefiting from the promotion of the PLUE protocol in mainland China, more than half of RTs can monitor LUS before and after prone positioning and 26.9% (88/327) can use the BLUE procedure. In addition, LUS was mainly used for diagnosing and monitoring already known or suspected lung diseases in previous reports, and the use was infrequent [25]. However, our study revealed that nearly half of RTs opted for daily routine screening. Some results were good, but others were not so good. The sinusoid sign, tissue-like sign, and quad sign were recognizable by a small percentage of participants and 30.6%(100/327) RTs without protocol; 74.6%(244/327) RTs could not calculate LUS scores. These findings suggested that RTs in mainland China lacked systematic LUS learning and standardized LUS practice. We will refer to the latest consensus, formulate clear and transparent LUS standards, and further expand standardized training in the future [3, 39].

Based on the survey results, most RTs sometimes or frequently changed their patient management strategies, and most of these changes were accepted by the physicians. This result was consistent with those of other studies, reflecting the clinical value of LUS [40, 41]. Assessing the respiratory muscles, especially the diaphragm, is important for diagnosing dyspnea and weaning failure [42]. Diaphragmatic ultrasound is a reproducible, accurate, and noninvasive technique that can be used in different work settings. Further, 61.2% (200/327) participants indicated that diaphragmatic inspiratory excursion, thickness of the diaphragm, and thickening fraction were used to determine the presence of diaphragmatic dysfunction. Ultrasound-guided pleural effusion drainage is a core skill of critical ultrasound; it was encouraging that more than 60% of the participants said that they did so [14]. Several studies recommended the use of ultrasound-assisted percutaneous tracheotomy, which was attempted in20.2% (66/327) RTs in our survey [43].

Moreover, 92.9%(459/494) of the participants said that the RTs should master echocardiography; even nearly 40% of the participants had already received echocardiography training. This might be because many patients had respiratory failure or difficulty weaning with cardiogenic risk [37, 44, 45]. Whether RTs should be trained on echocardiography and other point-of-care ultrasounds needs to be discussed cautiously.

### Limitations

Regarding the application and training of LUS received by the RTs, we collected the subjective feelings of the RTs. However, we did not ensure that each question was answered accurately and did not collect feedback from doctors or nurses on this. In addition, as a cross-sectional questionnaire, although we formulated relevant questions as detailed as possible, it was difficult for us to collect the specific practice steps and changing trends of the RTs implementing LUS. All of these might have led to deviations from actual conditions.

## Conclusions

The survey showed that, in mainland China, although the number and quality of the RTs have increased to a certain extent, the number is still small and the development is uneven across regions. Therefore, we need to promote and expand the number and workplace of RTs in mainland China. The value of LUS and the importance of training have been strongly subjectively recognized by RTs; many RTs have begun to apply LUS in clinical practice, even among patients with COVID-19. However, the data showed that RTs still needed a better understanding of LUS and many clinical limitations needed to be addressed. We need to further standardize the application of LUS practice and training for RTs in mainland China and establish corresponding certification pathways in the future. We should also call on doctors and RTs to work together, thereby removing relevant barriers and providing more support for the majority of patients with respiratory failure.

## Supplementary Information

**Additional file 1.**

## Data Availability

The datasets used and/or analyzed in the present study are available from the corresponding author on reasonable request.

## Abbreviations

CCUSG: China Critical Ultrasound Research Group
RT: Respiratory therapist
SBT: Spontaneous breathing trial
ICU: Intensive care unit
LUS: Lung ultrasound
NICU: Neonatal intensive care unit
PICU: Pediatric intensive care unit
COVID-19: Coronavirus Disease 2019
