# Supplementary material for: A cross-sectional survey on the lung ultrasound training and practice of respiratory therapists in mainland China
Source: BMC Pulm Med. 2022 Nov 18;22:425. doi: 10.1186/s12890-022-02213-6 (PMC9672549; doi:10.1186/s12890-022-02213-6)
Supplement: Supplementary file 1 — Additional file 1. [file 12890_2022_2213_MOESM1_ESM.docx]

**Addition file 1.0**

**A cross-sectional survey on the lung ultrasound training and practice of respiratory therapists (LUS-RT) in mainland China**

**(This questionnaire is for respiratory therapists only)**

1. **Demographic characteristics and basic information**
2. Name:
3. Age, yr:
4. Gender:

Male, female

1. The highest degree of education:

Associate degree, bachelor’s degree, master’s degree, doctor’s degree

1. Job ranking:

No job rank, level I, level II, level III, level IV

1. Years of working as an RT, yr:
2. Hospital name:
3. Location of hospital:
4. Hospital-level:

Level I, level II, level III

1. Department:

Respiratory care, respiratory/pulmonary, ICU, PICU, NICU, emergency, others

1. Number of the ultrasound machines in the department:

0, 1, 2, 3, 4, ≥5

1. Types of probes in department (multiple choice question):

Curvilinear probe, linear probe, phased array probe, transesophageal ultrasound probe

1. **Basic information of LUS training and practice**
2. Do you think the RTs need to learn LUS?

Yes, No

1. Have you received LUS training?

No training or simple training, in hospital training, in other hospital training, special LUS training course

1. Personal rating of the level of mastery and application of LUS?

Incapable, poor, fair or average, good or excellent

1. Are you willing to receive special training in LUS?

Yes, No

1. Can your department charge and report for LUS?

Yes, No

1. The frequency of applying LUS in your clinical work:

Never(1/month), rarely(1/week), sometimes(1/day), frequently(5/day)

1. the cause of limiting your use of LUS (multiple choice question)

Lack of proficiency, lack of time, lack of machine, lack of charges, lack of trust from clinicians

1. Have you ever applied LUS in COVID-19 patients?

No management, NO, Yes

1. Do you pay attention to LUS related research and papers?

Yes, No

1. **LUS practice details**

**(**For experienced RTs only. Participants were treated as experienced RT if they didn’t choose “never” when answering Q18 “The frequency of applying LUS in your clinical work?” in section 2.**)**

1. Which probes are commonly used for LUS (multiple choice question)

Curvilinear probe, linear probe, phased array probe

1. When to use LUS (multiple choice question):

Daily routine, dyspnea, hypoxemia, before and after the application of high-flow oxygen therapy (HFNC), before and after the application of non-invasive ventilation (NIV), before and after the intubation, before and after the tracheostomy, before and after SBT or extubation, before and after the fiber bronchoscopy (FOB), before and after chest physiotherapy (CPT), before and after prone position.

1. Can you identify these LUS signs (multiple choice question)?

Bat sigs, pleural sliding, A-line, B-line, Z-line, seashore sign, lung pulse, quad sign, sinusoid sign, stratospheric signs, lung point, shred sign, tissue-like signs, curtain sign, dynamic air bronchogram, static air bronchogram

1. Can you apply the BLUE protocol to the application of LUS?

Yes, No

1. Other LUS protocols that you are using (multiple choice question)?

Eight Zone Examination, Twelve Zone Examination, Twenty-eight Zone Examination, PLUE Protocol (prone position), No Protocol

1. Can you evaluate different LUS regions by semi-quantitatively score?

Yes, No

1. Do you change the respiratory therapy strategy according to LUS results?

Never, rarely, sometimes, frequently

1. Does the clinician approve of your change according to LUS results?

Reject, rarely accept, sometimes or mostly accept, completely accept

1. Will you issue an official report on the results of LUS?

Yes, No

1. Do you evaluate diaphragmatic dysfunction by diaphragmatic ultrasound (eg. diaphragmatic inspiratory excursion, thickness of diaphragm, and thickening fraction)?

Yes, No

1. Do you perform ultrasound-assisted tracheotomy?

Yes, No

1. Do you perform ultrasound-assisted chest drainage?

Yes, No

1. **Other ultrasound training and practice**
2. Do you think the RTs need to learn cardiac ultrasound?
3. Have you received cardiac ultrasound training?

Yes, No

1. Have you mastered and applied other ultrasound (multiple choice question)

No, Yes: cerebral ultrasound, kidney ultrasound, transesophageal ultrasound, ultrasound-guided invasive operation (arterial catheterization, deep vein catheterization, etc.)

**(Thank you for your support and participation!)**
